# Integrating clinician support with intervention design as part of a programme testing stratified care for musculoskeletal pain in general practice

**DOI:** 10.1186/s12875-021-01507-8

**Published:** 2021-07-26

**Authors:** Joanne Protheroe, Benjamin Saunders, Jonathan C. Hill, Adrian Chudyk, Nadine E. Foster, Bernadette Bartlam, Simon Wathall, Vincent Cooper

**Affiliations:** 1 Primary Care Centre Versus Arthritis, Keele School of Medicine, Keele University, Staffordshire, ST5 5BG UK; 2 STARS Research and Education Alliance, Surgical Treatment and Rehabilitation Service, The University of Queensland and Metro North Hospital and Health Service, Brisbane, QLD Australia

**Keywords:** Clinician support, Intervention development and testing, Stratified care, General practice, Musculoskeletal pain

## Abstract

**Background:**

Stratified care involves subgrouping patients based on key characteristics, e.g. prognostic risk, and matching these subgroups to early treatment options. The STarT-MSK programme developed and tested a new stratified primary care intervention for patients with common musculoskeletal (MSK) conditions in general practice. Stratified care involves changing General Practitioners’ (GPs) behaviour, away from the current ‘stepped’ care approach to identifying early treatment options matched to patients’ risk of persistent pain. Changing healthcare practice is challenging, and to aid the successful delivery of stratified care, education and support for GPs was required. This paper details the iterative development of a clinician support package throughout the lifespan of the programme, to support GPs in delivering the stratified care intervention. We argue that clinician support is a crucial aspect of the intervention itself, which is often overlooked.

**Methods:**

Qualitative research with patients and GPs identified barriers and facilitators to the adoption of stratified care, which were mapped onto the Theoretical Domains Framework (TDF). Identified domains were ‘translated’ into an educational paradigm, and an initial version of the support package developed. This was further refined following a feasibility and pilot RCT, and a finalised support package was developed for the main RCT.

**Results:**

The clinician support package comprised face-to-face sessions combining adult-learning principles with behaviour change theory in a multimethod approach, which included group discussion, simulated consultations, patient vignettes and model consultation videos. Structured support for GPs was crucial to facilitate fidelity and, ultimately, a successful trial. Clinician support is a two-way process– the study team can learn from and adapt to specific local factors and issues not previously identified. The support from senior clinicians was required to ensure ‘buy in’. Monitoring of GP performance, provision of regular feedback and remedial support are important aspects of effective clinician support.

**Conclusion:**

Designing effective clinician support from the onset of trial intervention design, in an evidence-based, theory-informed manner, is crucial to encourage active engagement and intervention fidelity within the trial, enabling the delivery of a robust and reliable proof-of-principle trial. We offer practical recommendations for future general practice interventions.

## Background

The STarT Back model of stratified care for low back pain (LBP) (stratifying care according to each patient’s risk of persistent disabling pain) has been adopted widely, following a proof-of-principle randomised trial in community physiotherapy clinics [1] and an implementation study in general practice [2]. A further programme of work, STarT MSK (Subgrouping for Targeted Treatment in MuSculosKetal conditions), has been developed to test stratified care for a broader range of patients with musculoskeletal (MSK) pain in general practice. Conducting a proof-of-principle trial in general practice is challenging but has the advantage that the intervention is simultaneously “road tested” in the target clinical setting, with elements designed in an “implementation-ready” manner, enabling rapid translation into clinical practice.

General practice is a complex environment and, currently in the UK (before and since the COVID-19 pandemic), is under unprecedented pressure. In part, this is due to a shortage of general practitioners (GPs) and an ageing population, with high levels of multi-morbidity, where management is not simply a sum of the parts [3]. Changing healthcare practice in such circumstances is challenging [4–6]. Important considerations are clinicians’ preference for established frameworks, whether they consider the intervention meaningful and relevant [7] and perceived threats to clinical autonomy [8]. Failure to address such concerns in clinical trials that are focused on innovation can result in poor recruitment to trials, compounding poor or misleading data, and a risk that the intervention will not be adopted [9].

In clinical practice, clinical decision support systems are most effective when combined with education for the professionals using them [10] and the perceived usefulness of the interventions is a decisive factor in their acceptance by clinicians [11]. In the research domain, there is a risk of focusing exclusively on research design and delivery, with clinician education and support an afterthought. In this research programme, it was clear from the start that a thorough design process would be needed for the stratified care intervention for MSK pain to be used effectively and without disrupting the consultation, and that the simultaneous design of a fully integrated clinician support package would be required. The multi-disciplinary research team brought expertise from general practice, physiotherapy, clinical research, epidemiology, social sciences, health informatics and medical education.

The aim of this paper is to detail the steps in developing and integrating a clinician support package to support GP engagement in delivering the STarT MSK intervention. We draw together findings from other parts of the programme – which have previously been published in this journal – and outline how these findings were ‘translated’ into an educational paradigm to inform the iterative development of clinician support from the initial stages of the 6-year programme through to the stage of being ready to test within a full RCT. We argue that developing adequate clinician support is a crucial aspect of broader intervention development, which is often overlooked during broader clinical intervention development, and we provide practical recommendations that can serve as a guide for the development of future primary care interventions.

### The STarT MSK research programme

The development of the clinician support package described here was conducted within the context of the 6-year STarT MSK programme consisting of four work packages (see Table 1, below). Several of the studies from these work packages have previously been published in this journal [4, 15, 16]. Final results of the clinical trial are yet to be published.

**Table 1:** Outline of the four STarT MSK research programme work packages

• WP1 – epidemiological study to test and validate the Keele STarT MSK tool to predict patients’ risk of persistent pain [12]
• WP2
◦ Qualitative research with patients and clinicians to identify anticipated barriers and facilitators to the adoption of stratified care [4]
◦ Evidence synthesis of effective treatments [13]
◦ Consensus process with stakeholders and practitioners to agree recommended matched treatment options for patients at low, medium and high risk, for decision-making in general practice [14]
◦ Integrated delivery platform within the electronic health record (EHR)
◦ Development of clinician support package
• WP3 – pilot/feasibility cluster randomised controlled trial of stratified care in general practice [15, 16]
• WP4 – main cluster randomised controlled trial of stratified care in general practice [17]

The stratified care intervention is described in detail elsewhere [12, 17] and comprises two components: the use of the prognostic risk tool (Keele STarT MSK Tool) and the selection of an appropriate clinical treatment, matched to the patient’s prognostic risk group. In brief, the key components for intervention practices were:

- A computer template within the electronic health record (EHR), triggered automatically on entering a relevant MSK diagnosis or symptom into the patient’s EHR, asking the GP to complete the Keele STarT MSK Tool, based on patients’ responses to ten prognostic questions
- Automatic calculation of the patient score as being of high, medium or low risk of persistent pain
- Presentation to the GP of recommended matched treatment options for the patient, based on pain site (e.g. back, neck, knee, shoulder or multi-site) and risk subgroup
- Integration of self-management information resources to be shared with the patient

Within the MSK consultation, the key objectives were:

- To ensure that the GP engages with the EHR to trigger the stratified care template whilst the patient is still present
- For the GP to use the risk stratification tool and to discuss the matched treatment options with the patient before agreeing a management plan
- For the GP to feel confident in being able to integrate the stratified care intervention with their usual clinical history taking, examination, reasoning and diagnosis.

GPs in control practices, within the cluster randomised controlled trial, were required to continue “usual care”, after completing a brief template to identify eligible control participants to be invited into the trial. Patient recruitment to the STarT MSK trial reached its target in July 2019, after 14 months. Follow-up measures were completed in Feb 2020 and the trial data is currently being analysed. The results of the trial are anticipated during 2021.

In what follows we outline the methods used in iteratively developing, piloting and revising the clinician support package, before presenting the results of the finalised support package that was delivered as part of the main RCT.

## Methods

### Designing the components of the clinician support package

Clinician support was developed through an iterative approach, using theory and methods from both social science and medical education disciplines. The pilot version of the clinician support package was initially developed throughout work packages 1 and 2, as summarised in Table 2, below.

**Table 2:** Development of clinician support throughout work packages 1 and 2 of the STarT-MSK research programme

**Programme Phase**	**Programme activity**	**Clinician support package**
WP1	• Epidemiological study • Tool development and validation	• Information about what questions would be asked during the consultation
WP2 Exploratory phase	• Focus groups with GPs and patients. • Analysis using Theoretical Domains Framework (TDF)	• Perceptions of GPs and patients and identification of possible barriers and facilitators to the adoption of stratified care, mapped onto the TDF and onto specific behaviour change techniques
WP2 developing the intervention	• Evidence synthesis and expert consensus groups to devise evidence-based matched treatments	• Identification of training/support issues relating to matched treatments and how they are presented in the Electronic Health Record (EHR) tool
	• Development of the interactive tool for the EHR	• Influencing tool design to address issues identified in qualitative work • Identification of training issues related to the IT and integration into consultations
	• Refinement and testing of the electronic tool	• “Translation” of qualitative findings and experience of intervention design into educational paradigm to inform support package • Design and testing of educational input and support materials • User testing of the proposed EHR template with GPs • Planning training sessions

### Underpinning research

Development of the STarT MSK intervention itself had begun, with exploratory focus groups and individual interviews with GPs and patients, which identified barriers and facilitators to the adoption of stratified care, giving rich insights into the environment, pressures, motivations, beliefs and expectations of both groups, and providing an understanding of the acceptability of using stratified care and how it might fit within GP consultations [4]. The findings represented the core data used to inform development of both the StarT MSK intervention and the clinician support package.

A theoretically underpinned approach to analysis enabled a robust and coherent explanation of the findings. The Theoretical Domains Framework (TDF) [18] was employed, a framework that synthesises 112 psychological constructs determining behaviour change into fourteen domains including knowledge, skills, social/professional role and identity, memory, attention and decision processes. It facilitates identification of barriers and facilitators of clinical behaviour change at an individual and organisational level. The themes developed through analysis of the focus group and interview data were mapped onto the TDF domains, in order to explore the degree to which the identified themes could be seen to ‘fit’ within these domains, and how the theoretical constructs manifested in relation to these themes. This allowed for identification of key theoretical domains to target for clinical behaviour change; including, *knowledge, skills, professional role and identity, environmental context and resources,* and *goals* (see [4] for full description). The identified domains informed development of the intervention format and provided a starting point for developing the clinician support package.

In parallel to this work, we began to design the platform to deliver the Keele STarT MSK tool and matched treatment recommendations within the EMIS Web clinical EHR, used by many UK GPs and all participating practices. EMIS allows bespoke protocols and data entry templates to be designed then implemented in target practices. We designed a version of the tool to be embedded within the system for use during face-to-face consultations. This had to meet both the needs of the research and requirements of the user, i.e. complement the consultation, be easy to use in a time pressured environment and record clinical information in a meaningful way for future use.

### Further analysis: translation of theoretical domains into an educational paradigm

The theoretical domains relevant to GP behaviour change that were identified from the qualitative research fell short of specifying an educational and support package. This required further “translation” of these strategies into an educational paradigm, using well-recognised adult learning principles [19, 20]. A modified form of Bloom’s taxonomy of learning [21] suggests division into three domains of learning: cognitive, affective and psychomotor, commonly represented in the form of knowledge, skills and attitudes. The importance of this approach is that educational activities can be aligned [22] to the learners’ needs (barriers and facilitators identified in the TDF) and to intended outcomes (engagement with and use of the tool and matched treatments).

In designing educational interventions, there is a risk of privileging “delivering” knowledge – what the teacher/researcher needs to “tell”, rather than what the learner/GP needs to know. Using the three domains as a framework ensures particularly that affective components, e.g. beliefs, motivation, doubts and difficulties are addressed adequately and that skills components are taught appropriately.

#### Affective domain

We realised that, for intervention practices, this study would have a significant impact on GP consultations (i.e. completing the prognostic tool and accessing recommended matched treatment options) which may differ from GPs’ usual practice. The qualitative findings indicated that GPs’ beliefs about the validity, worth and feasibility of the approach would be important elements in the affective domain. This suggested that a comprehensive educational package was needed, ideally split into two sessions, beginning with discussion amongst GPs about how they consult and make decisions. There should be an opportunity to try the tool, reflect on its use and share perceptions and experiences with colleagues. Leaving practices with “dummy” patients and a trial system during the interval between sessions would allow experiment and stimulate questions and discussion at the next session.

#### Cognitive domain

GPs would also need some knowledge of the principles of stratified care and how it differs from the usual ‘stepped’ care approach, the Keele STarT MSK tool and its derivation, the matched treatments and essential elements of the trial design and conduct. Inclusion of background information about previous studies and the study team might also contribute positively. This cognitive component could be provided by a didactic/interactive group session with slides and printed material.

#### Psychomotor domain

The psychomotor or skills domain includes the use of the tool within the EHR and some complex consultation skills elements:

- interacting with a computer earlier in the consultation than is usual for most GPs, in order to launch the tool
- explaining to patients the use of the tool and decision support element
- integrating these elements into the consultation at appropriate stages
- managing the consultation within allocated time slots

Skills development requires active participation and we designed a simple consultation simulation, using a vignette of a typical patient, for GPs in each practice to experiment and discuss approaches to integrating the tool. The design team had ideas about the “best fit” of the tool and matched treatments in a consultation but were not prescriptive and learned from observation of, and discussion with, GPs in training sessions. This allowed more guidance to be provided in the main trial. Performance monitoring and feedback are important elements for encouraging engagement and we included a system for collating basic performance data at individual GP, practice and trial arm level for anonymised feedback during an early review visit to each practice.

### Reviewing clinician support in the pilot trial

The iterative development work throughout the initial phases of the programme led to development of a package to support GPs in delivering stratified care as part of the pilot RCT. The pilot RCT tested the STarT MSK intervention in eight GP practices (four stratified care practices; four control practices). Full details of the pilot RCT have been published previously in this journal [15, 16]. Table 3, below gives an overview of the clinician support package; Table 4 provides full details of the structure and content of the training delivered to GPs as part of the pilot trial:

**Table 3:** Outline of pilot clinician support package

Clinician support in pilot study
Overall scope and plan
Total training time available to GPs is 4 h, provisionally to be in two 2 h sessions. Optionally, this can be supplemented by one “catch up” session with individual GPs at their request or in response to problems identified by the study team
Two TAPS facilitators to attend each session, aiming at continuity of at least one for both sessions
Training approach
Training is for individual practices and based on all GPs attending both sessions and working as a small group with the Keele GP facilitators. There are some knowledge and skills components to be covered and the entire sessions should be interactive and collaborative, exploring and building on the GPs’ current practice. Particularly during the pilot phase, there will be lessons for the study team to learn and, possibly, some changes to be made to the intervention, so the facilitators will gather information for the team as well as delivering and documenting the training
Key issues to address
• Tool complements normal clinical practice and does not replace it
• It is a prognostic tool to aid management, not a diagnostic tool
• A key step in integrating the tool into the consultation – is the need to enter a provisional Read code *during* the consultation to trigger the template
Requirements for delivering the training
• Protected time for all GPs to attend
• Co-ordination with practice manager
• Training room suitable for small group learning
• Computer, linked to clinical system, with display visible to the group
• TAPS templates installed and tested
Support Materials
• Slide sets for sessions 1 and 2
• Patient vignettes from TAPS
• Laminated copy of STarT MSK tool and matched treatment options
• Plan and record for training sessions to complete at each practice

**Table 4:** Details of structure and content of pilot GP training sessions

**Timing**	**Topic**	**Detail**	**Methods & *Resources***
**Session 1**			
10 Min	Introductions	▪ Personal introductions, roles, etc. ▪ Brief outline of the practice and its population ▪ Special interests of GPs	*Pre-trial background sheet completed by practice* ▪ Informal chat to get people warmed up
10 Min	Brief outline of study, its background and scope	▪ Origins of research in STarT Back ▪ Explain prognostic risk ▪ Clinical conditions and sites involved ▪ What we are investigating, in general terms	*Few slides – scant detail* ▪ Interactive presentation and brief Q/A
10 Min	GPs’ current management of these conditions	▪ Diagnostic approaches – bio-mechanical/ bio-psycho-social – use shoulder pain as example ▪ Investigations routinely used – what and where? ▪ Advice generally given to these patients ▪ Sickness certification ▪ Medication preferences and usage ▪ Physiotherapy etc availability and usage ▪ Referral options and patterns for different pain sites – MSK, surgical etc ▪ Significant constraints they experience ▪ Patients’ expectations – e.g. Imaging, certificates, referral	*Pre-trial background sheet* ▪ General discussion to gauge GPs’ philosophy and general approaches – helps build relationship and aid to tailoring our approach to training ▪ Avoid detail on specific conditions within MSK *Flip chart to explore treatment/referral options for the practice*
20 Min	GPs’ usual consultation habits	▪ Map out their usual consultation process/flow ▪ Is computer used during or after consultations? ▪ Read coded diagnosis entered at provisional stage or not ▪ Any existing use of templates and decision aids? ▪ Use of interactive tool plus printed advice eg PILS	▪ More informal discussion *A4 sheet with a few prompt statements for GPs* *Pads of paper for GPs’ notes* *Sticky notes pads to capture notes and queries for later*
20 Min	Stratified care approach	▪ What is stratified care and how does it differ? ▪ Why it may have advantages for patients and NHS ▪ Basis for prognostic stratification tool ▪ Expected proportion in each risk group ▪ The tool identifies potential treatment targets ▪ How this complements usual diagnostic clinical practice ▪ Matched treatment options and how we devised them ▪ No change in local pathways during the study – treatment options are pointers to be used with these pathways	▪ Interactive presentation and Q/A *Slides:* *Knowledge about stratified care* *Establish credibility of tool and matched treatments* *Emphasise “Risk” is of chronicity/complexity not pathology* *Explain complementarity with diagnostic process* *No new pathways at this stage*
45 Min	The STarT MSK tool in practice	▪ Overview of questionnaire and matched treatments ▪ Key GP behaviours the tool tries to nudge/change ▪ Providing the tool score to onward treating clinicians ▪ Trying out the tool – paper exercise: ▪ GPs work in pairs, each with a vignette ▪ One asks questions and completes paper tool, other responds from vignette ▪ Swap roles for second vignette ▪ Compare scores and experience of using tool ▪ Demonstration of integrated template by facilitator ▪ All GPs trying it out with support	▪ Discussion around *slides:* *Pyramid slide for overview* *Questionnaire and matched treatments* ▪ Giving patients score and recommended options Communicating score in referrals *Paper copies of vignettes and risk tool* *Live EMIS system with template* ▪ Demo of template use ▪ All GPs trying out template, using vignettes, with no attempt at consultation elements *Vignettes needed: Low risk knee pain, Medium risk shoulder pain, High risk multisite pain with co-morbidity*
5 Min	Suggested preparation for Session 2	▪ Try template a few more times with dummy patients ▪ Look at treatment options and linked patient info	▪ Replace this with a short break if running 2 sessions together – *would need refreshments*
**Session 2**			
10 Min	Reflections from Session 1	▪ Questions about stratified care concept ▪ Feedback from trying out tool ▪ Practical issues and any doubts	▪ Reminder of key elements we covered in Session 1 ▪ Discussion of any issues ▪ Skip if running 2 sessions together
60 Min	Simulated “consultations” using vignettes	▪ GP or one of team gives outline from a TAPS vignette, as a patient might present ▪ What to say to the patient about the tool and risk groups ▪ GP uses template to get score and treatment options ▪ GP explains and negotiates options ▪ Facilitator might try asking/challenging for other options ▪ Each GP has at least one turn at simulation	▪ Skills session ▪ Emphasise simulation and not role play ▪ Use selection of low/medium/high risk vignettes as basis *Set up clinical computer in a consulting room if possible* and run as a consultation, each taking a turn ▪ GP or facilitator gives outline story ▪ Facilitator can present challenges for consulting GP ▪ Group works together on suggestions – problem-solving approach *Prompt sheet for consultations*
10 Min	Discussion of simulated consultations	▪ GPs’ belief and trust in score and recommendations ▪ Practicalities of negotiating recommendations with patients ▪ Dealing with inappropriate demands	▪ Discussion to explore beliefs and confidence in approach and tools, having had the experience ▪ Anticipated challenges and how to handle them
15 Min	Diagnostic issues and priorities vs stratification options	▪ Discussion about complementarity of clinical diagnosis and prognostic stratification ▪ Examples of “clinical override” of risk stratification	▪ Discussion *Few clinical vignettes to illustrate situations where clinical diagnosis or situation might take precedence, eg:* *PH of breast/prostate cancer* *Chronic problem with many failed treatment attempts* *Frailty/multi-morbidity*
10 Min	GP management of low risk patients	▪ Effective reassurance ▪ GPs’ confidence in managing low risk ▪ Resources available for low risk management ▪ Other primary care team members involved in low risk?	▪ Discussion about how GPs will manage low risk ▪ How to provide effective reassurance ▪ Look at advice materials *Printout of PILS + Leaflets*
10 Min	Management of medium and high risk patients	▪ Addition of layers to complement low risk management ▪ Directed at specific pathology and wider issues e.g. co-morbidity, psycho-social, employment, etc	▪ Discussion around recommended treatment options *Paper copies of matched treatments to illustrate*
5 Min	Action plan	▪ Dealing with queries ▪ Additional support if needed ▪ Who to contact etc	

We had already established that affective elements are crucially important to the success of the trial. Due to the complexity of the task, training for intervention practices was delivered by two members of the study team: one GP and one clinical researcher. Most or all GPs in intervention practices attended the training sessions, though a few attended only one of the two linked sessions. Overall, the training was delivered to time and to plan, and GPs participated actively in discussions and practical sessions. Facilitators from the local Clinical Research Network undertook the brief training for control practices in the pilot trial.

The Keele STarT MSK tool was designed so that all selections made on the template were coded within the patient’s medical record. Anonymised data were extracted from each participating practice on a regular basis to facilitate analysis of the tool’s usage at three levels: individual GP, single practice and trial arm. These data were an essential part of monitoring and encouraging GP engagement with the stratified care template. We could identify any under-performing practices or individual GPs at an early stage, intervene and work with them to identify and remedy potential barriers. Examples included GPs not entering clinical codes and thus not triggering the tool, and information about using the tool and matched treatments not being disseminated to new members of staff. On a monthly basis each practice received an audit report on their tool usage at individual GP level.

A key element of the tool was to enable GPs to opt out of using it whilst discouraging the use of the “Esc” key which would have left no further engagement data in the EHR. Instead, GPs opting-out selected reasons; for instance, ‘patient not appropriate’ or ‘no time to complete’, enabling the team to assess the feasibility of using the tool in consultations.

A nested qualitative study was conducted as part of the pilot trial, in which consultations were video or audio recorded and used to stimulate recall in post-consultation interviews with matched pairs of GPs and patients, to explore the acceptability and feasibility of delivering the stratified care intervention [16]. We found difficulties in integrating the intervention in consultations within the standard 10-min timeframe. However, GPs did report finding this easier with practice throughout the course of the pilot. This was a positive finding that provided us with some encouragement ahead of the full trial, particularly given the well-established challenges in bringing about GP behaviour change.

A frequent comment was that 4 h of GP training was excessive and difficult to accommodate. GPs valued the skills component but felt that they needed less information on the trial background and recommended that the training be reduced to a single 2-h session for the main trial. They also felt that the team should focus the training on how best to integrate the stratified care approach within a routine MSK consultation.

### Refinement of clinician support package

Recommendations from the pilot were incorporated into the support package and shortened training for the main trial. See Table 8, below, for a summary of the refinements made to the clinician support package based on pilot trial findings. As a result of direct observation of training simulations, discussion with participating GPs and the qualitative work [16], we also felt able to be more prescriptive about the best fit of the intervention within a consultation and produced a short video of a simulated consultation to illustrate this. Refinements made to the clinician support package are summarised in Table 5, below:

**Table 5:** Refinement of clinician support package following the learning in the pilot trial

**Problems identified in pilot**	**Action taken before main trial**
• Cumbersome questionnaire wording and variable use of terms	• Design and validation of specific clinical version, with constructs stated for GPs
• Sub-optimal treatment recommendations	• Rationalisation and refinement
• Excessive length of clinician support sessions and requests to re-focus some parts	• Reduced to one 2 h session with less background information
• Trainers reluctant to specify best fit of intervention within consultation	• Application of experience gained to be more directive, including production of video of simulated consultation for training
• Some GPs missed clinician support sessions	• Training logs and prompt sheets introduced for each practice
• Delays in detecting problems and taking remedial action with practices	• Early monitoring and re-visit to practice. • Monthly feedback and personal contact by same trainer
• Poor engagement and performance by control practices	• Control and intervention practices to have training visit from GP and clinical researcher from study team • More focused clinician support sessions for control practices

Whilst both GPs and patients felt the Keele STarT MSK tool added value to the consultation, some items, derived from the original self-administered tool in WP1, were judged to be “cumbersome” (See [16] for full details). This led to development and validation of a clinical version of the tool, with more conversationally styled questions; also including a statement of the construct underpinning each item to help clinicians effectively communicate these to patients [23]. Support materials for the main trial were updated to include the revised tool and the training programme was adjusted to focus on the item constructs and the need to adhere to the exact wording of questions.

From outcome measures in the pilot trial, in particular GP engagement measured through the proportion of relevant consultations in which GPs used the brief template to identify eligible control participants to be invited into the pilot (see [15] for full results), it was clear that the brief training for control practices was not engaging many GPs and posed a risk of attrition bias. For the main trial we decided to use the same GP and clinical researcher pairs for clinician support in both trial arms, with a 1-h session for control practices focused on the recruitment template and the purpose and importance of the research.

## Results

### Clinician support in the main trial

The finalised clinician support package comprised face-to-face sessions combining adult-learning principles with behaviour change theory in a multimethod approach, which included group discussion, simulated consultations, patient vignettes and model consultation videos. The clinician support package also included printed and laminated prompt sheets for GPs, a training log to ensure that no GPs were excluded, and a plan agreed with each practice to cascade the training to any new recruits or locum GPs.

Clinician support was delivered by the same team at all 24 practices (intervention and control) in the main trial. Almost all participants attended the single clinician support session and the level of understanding and engagement appeared strong, despite the shortened session. Table 6, below, gives an overview of the finalised clinician support package and Table 7 provides full details of the structure and content of the training delivered to GPs as part of the main trial:

**Table 6:** Outline of the finalised clinician support package

Clinician support for main trial
Overall scope and plan
Members of the TAPS team will provide training sessions for all participating GPs. For control practices, this will be a 1 h session. For intervention practices, this will be about 2 h. Optionally, this can be supplemented by one “catch up” session with individual GPs at their request or in response to problems identified by the study team.
Training for control practices will be by one team member. Ideally, two trainers will facilitate the more complex sessions for intervention practices.
Training approach
Training is for individual practices and based on all GPs attending and working as a small group with the Keele GP facilitators. There are some knowledge and skills components to be covered and the entire sessions should be interactive and collaborative
Requirements for delivering the training
• Protected time for all GPs to attend
• Co-ordination with practice manager
• Training room suitable for small group learning
• Computer, linked to clinical system, with display visible to the group
• TAPS templates installed and tested
Key issues to address for intervention practices
• Tool complements normal clinical practice and does not replace it
• It is a prognostic tool to aid management, not a diagnostic tool
• A key step in integrating the tool into the consultation – is the need to enter a provisional Read code *during* the consultation to trigger the template
• Arrangements for physiotherapy referral and liaison
Support Materials
• Slide sets for sessions 1 and 2 and single session for controls
• Patient vignettes from TAPS
• Laminated copy of STarT MSK tool and matched treatment options
• Plan and record for training sessions to complete at each practice
• Laminated prompt sheets for both intervention and control practices

**Table 7:** Details of structure and content of main trial GP training sessions

**Timing**	**Topic**	**Detail**	**Methods & *Resources***
**Intervention Practice – 2 h in a single session**			
10 Min	Introductions	▪ Personal introductions, roles, etc. ▪ Brief outline of the practice and its population ▪ Special interests of GPs	*Pre-trial background sheet completed by practice* ▪ Informal chat to get people warmed up
15 Min	Brief outline of study, stratified care approach and pilot study results	▪ Background to study ▪ Explain prognostic risk ▪ Clinical conditions and sites involved ▪ Summary of pilot results ▪ Proportion in each risk group	*Few slides – scant detail* ▪ Interactive ▪ *Emphasise “Risk” is of chronicity/complexity not pathology* ▪ *Explain complementarity with diagnostic process* presentation and brief Q/A
30 Min	The STarT MSK tool in practice	▪ Overview of questionnaire and matched treatments ▪ GP actions we hope to foster ▪ Providing the tool score to onward treating clinicians ▪ Trying out the tool – paper exercise: ➢ GPs work in pairs, each with a vignette ➢ One asks questions and completes paper tool, other responds from vignette ➢ Swap roles for second vignette ➢ Compare scores and experience of using tool ▪ Use of the tool in consultations - video	▪ Discussion around *slides:* *Pyramid slide for overview* *Questionnaire and matched treatments* ▪ Giving patients score and recommended options Communicating score in referrals *Paper copies of vignettes and risk tool* *Live EMIS system with template* ▪ Demo of template use ▪ All GPs trying out template, using vignettes, with no attempt at consultation elements *Video of mock TAPS consultation*
30 Min	Simulated “consultations” using vignettes	▪ Facilitator gives outline from a TAPS vignette, as a patient might present ▪ GP uses template to get score and treatment options ▪ GP explains and negotiates options ▪ Facilitator might try asking/challenging for other options	▪ Skills session ▪ Emphasise simulation and not role play ▪ Use selection of low/medium/high risk vignettes as basis *Set up clinical computer in a consulting room if possible* ▪ GP or facilitator gives outline story ▪ Group works together on suggestions – problem-solving approach *Prompt sheet for consultations*
10 Min	GP management of low risk patients	▪ Effective reassurance ▪ GPs’ confidence in managing low risk ▪ Resources available for low risk management ▪ Other primary care team members involved in low risk?	▪ Discussion about how GPs will manage low risk ▪ How to provide effective reassurance ▪ Look at advice materials *Printout of PILS + Leaflets*
20 Min	Management of medium and high risk patients	▪ Addition of layers to complement low risk management ▪ Directed at specific pathology and wider issues e.g. co-morbidity, psycho-social, employment, etc ▪ Physio hubs and provision we have negotiated ▪ Detail of physio referral process – how would GPs like us to set it up? ▪ Liaison with physio in high risk patients if needed – Email arrangements	▪ Discussion around recommended treatment options ▪ Emphasise MSK rehab for high risk ▪ Hub physios to attend if possible to build personal relationship and clarify arrangements *Paper copies of matched treatments to illustrate*
5 Min	Action plan and lead GP actions	▪ Lead GP role: ➢ Keep a training record ➢ Cascade training to locums, etc ➢ Respond to monthly feedback email ➢ Liaise with team over any issues or problems ➢ Dealing with queries ▪ Additional support if needed ▪ Who to contact etc	*Training record for practice* *Sample monthly feedback report* *Prompt sheet for GPs*
**Training session for control practices – 1 h or less**			
10 Min	Introductions	▪ Personal introductions, roles, etc. ▪ Brief outline of the practice and its population ▪ Special interests of GPs	*Pre-trial background sheet completed by practice* ▪ Informal chat to get people warmed up
10 Min	Brief outline of study its background and scope	▪ Clinical conditions and sites involved ▪ What we are investigating, in general terms ▪ Questionnaires to patients ▪ Medical record review	*Few slides – scant detail* ▪ Interactive presentation and brief Q/A
10 Min	What we ask of GPs	▪ Patient verbal consent for study and record of this ▪ Pain score and pain site recorded in >50% ▪ Usual care of patients	*One slide*
15 Min	EMIS template	▪ Demonstration of real template and practice with it	
5 Min	Additional support	▪ Coping with GPs absent from training or joining later Briefing session by Practice Manager	*Laminated prompt sheets for all GPs*

### Monitoring and feedback

Monitoring and feedback are essential components of an educational process and, as such, a major change from the pilot trial was the availability of performance data for practices and individual clinicians throughout the main trial. Through weekly data extraction from all practices and monthly analysis and reporting, the study team monitored activity and performance of practices and individual GPs in the trial. This demonstrated a high level of engagement with the tool and use of recommended matched treatment options according to risk stratification of individual patients and these data will be reported in the trial results paper to be published later this year.

**Fig. 1:**
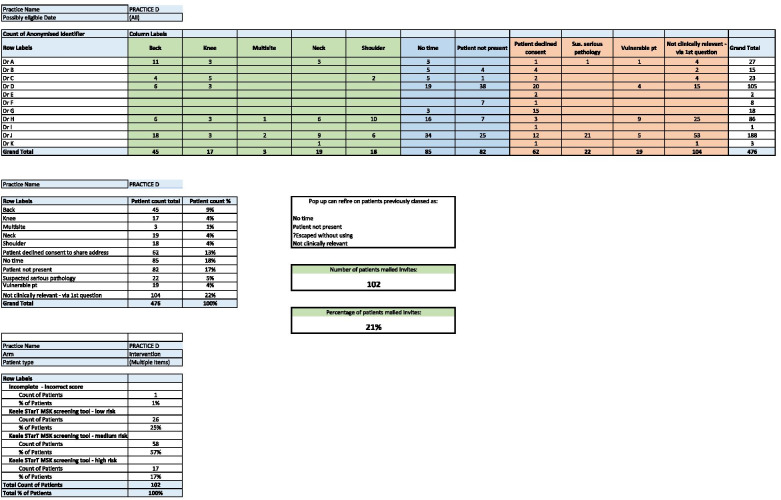
Sample of feedback data to intervention practices

Each month, the same study team member emailed the lead GP and practice manager, including the performance table (Fig. 1), as feedback and for motivation.

Where specific problems were identified or overall performance was declining, the study team member assigned to that practice would make contact, initially by phone and arrange further support to an individual or to the practice, either by phone or a visit to the practice. This might also involve our informatics specialist if technical issues were identified. Commonly, performance problems related to new or returning GPs and a failure to cascade the training effectively within a practice.

## Discussion

This paper has detailed the iterative development throughout the course of a large-scale programme, of clinician support to deliver a complex intervention in the context of an RCT, and as such, can be a valuable practical resource for researchers developing future primary care interventions. There is a wealth of literature on the development of theory-informed interventions that aim to change clinician behaviour, with varying results (see, for instance: [24–27]). Much of this literature focuses on development of behaviour change interventions themselves, and on evaluating clinicians’ and patients’ uptake and engagement [28–30]; and this research has highlighted some similar challenges to those identified here, including the practical and time issues involved in engaging clinicians, and the challenge of achieving clinician ‘buy in’. There has been a lack of focus in the literature, however, on specifically developing the clinician support to enable successful delivery of interventions in RCTs, as reported here, which we argue is a crucial element of intervention development that is often overlooked.

Additionally, whilst other studies have commonly drawn on behaviour change theory such as the TDF and COM-B models to inform clinical intervention development, only very few have combined this with adult learning theory. Porcheret et al. [31] developed an intervention to be delivered by GPs to enhance self-management support for people with osteoarthritis (OA) in primary care, using the TDF to identify relevant behaviour change domains, and adopted adult learning theory to decide on the style of delivery of the intervention. However, the focus was on how the OA intervention could best be delivered in consultations to support patients’ learning, rather than the learning of GPs. Likewise, Gallagher and Bell [32] discuss the use of adult learning as part of an occupational therapy intervention for bladder and bowel management after spinal cord injury, but again the use of adult learning is focused on the learning by the patient, rather than in supporting the learning of the clinicians themselves to deliver the intervention, as we did in the current programme. This paper is therefore novel in its focus on clinician support as a key element of intervention design.

### Strengths and limitations

It is both a strength and limitation of the development of this clinical support intervention that no entirely new data was collected. Instead, the focus was on building upon data collected for the STarT MSK intervention and mapping it onto established adult learning theory. To our knowledge this has not been previously reported as a method to support GP behaviour change in the context of a complex RCT. This is crucial, especially in a proof-of-principle trial, because if the intervention is not fully adopted within the trial the results will be less reliable. It is a limitation that we did not gather new data specific to the development of the clinical support package, however, we argue that this may have been additionally burdensome to the GPs and practice staff recruited to the programme of research.

### Recommendations for future interventions in general practice

In describing the design, refinement and delivery of the intervention and clinician support package, this paper emphasises the importance of an integrated approach and of incorporating robust educational principles and practice (See Table 8).

**Table 8:** Use of educational theory in developing clinician support

**Educational component**	**Detail**
Educational needs assessment	▪ Focus groups, TDF, feedback from GPs on pilot training
Task analysis	▪ Components identified as Cognitive (knowledge), Affective (attitudinal) or Psychomotor (skills) domains
Constructive alignment	▪ Selection of appropriate methods, tools and content to address domains, particularly clinical and IT skills
Session planning	▪ Length of training, balance of components, methods and resources. Selection and preparation of trainers
Delivery of training	▪ Sessions booked and delivered at practices
Records and “safety net”	▪ Training log and plan to train any who miss session
Monitoring of performance	▪ Regular data extraction and analysis
Feedback	▪ Early intervention if problems identified ▪ Monthly email feedback with performance data and encouragement
Evaluation	▪ Qualitative interviews with a sample of GPs

We make the following recommendations for integrating systematic clinician support into future complex interventions in general practice:

1. General practice is a complex and pressured environment, so the potential impact of an intervention must be anticipated and explored thoroughly.
2. Structured support is crucial to enabling fidelity and, ultimately, a successful clinical trial.
3. Clinician support is a two-way process – the study team can learn from and adapt to specific local factors and to issues they have not previously identified.
4. Professional identities are important – support needs to be from senior clinicians, perceived as understanding the task and pressures involved.
5. Monitoring of performance matters, as does early intervention if problems appear.
6. Feedback on performance is a key element in support.

## Conclusion

This paper has described and demonstrated the importance of designing clinician support in an evidence based, theory informed manner, in this case combining the qualitative findings using the Theoretical Domains Framework (TDF), with adult learning principles [19, 20]. Effective clinician support enables active engagement and intervention fidelity within the trial, enabling the delivery of a robust and reliable proof-of-principle trial. We have argued that developing adequate clinician support should be viewed as an important aspect of broader intervention development, which is considered from the onset of intervention design, and not only as an add-on. We intend for the practical recommendations we have provided to serve as useful a guide for the development of future primary care interventions.

## Data Availability

In line with the Standard Operating Procedures in place at the School of Medicine, where this study was conducted, data are archived at a dedicated location within the Keele University’s network. A request to access archived data can be made by completion of a Data Transfer Request form, which can be accessed by contacting: Primary Care Centre Versus Arthritis, School of Medicine, Keele University, Staffordshire, ST5 5BG, UK; Tel: +44 (0) 1782 733905.

## Abbreviations

GP: General practitioner/ general practice
RCT: Randomised controlled trial
TDF: Theoretical Domains Framework
NHS: National Health Service
EHR: Electronic Health Record
